# Ethyl 2-(3,4-dimethoxy­benz­yl)-1-phenyl­sulfonyl-1*H*-indole-3-carboxyl­ate

**DOI:** 10.1107/S1600536809026506

**Published:** 2009-07-15

**Authors:** B. Gunasekaran, Radhakrishnan Sureshbabu, A. K. Mohanakrishnan, G. Chakkaravarthi, V. Manivannan

**Affiliations:** a Department of Physics, AMET University, Kanathur, Chennai 603 112, India; b Department of Organic Chemistry, University of Madras, Guindy Campus, Chennai 600 025, India; c Department of Physics, CPCL Polytechnic College, Chennai 600 068, India; d Department of Research and Development, PRIST University, Vallam, Thanjavur 613 403, Tamil Nadu, India

## Abstract

In the title compound, C_26_H_25_NO_6_S, the phenyl ring forms a dihedral angle of 82.5 (1)° with the indole ring system. The mol­ecular structure is stabilized by weak intra­molecular C—H⋯O inter­actions and the crystal structure is stabilized by weak inter­molecular C—H⋯O inter­actions.

## Related literature

For the biological activity of indoles see: Macor *et al.* (1992 ▶); Williams *et al.* (1993 ▶); For related structures, see: Chakkaravarthi *et al.* (2007 ▶, 2008 ▶). For graph set notation see: Bernstein *et al.* (1995 ▶).
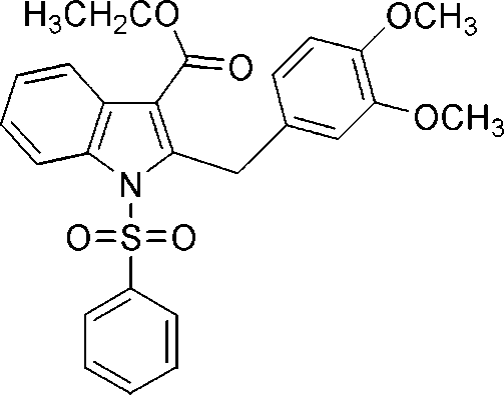


## Experimental

### 

#### Crystal data

C_26_H_25_NO_6_S
*M* *_r_* = 479.53
Triclinic, 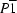

*a* = 9.2914 (3) Å
*b* = 9.3008 (3) Å
*c* = 14.1561 (5) Å
α = 87.367 (2)°
β = 76.158 (2)°
γ = 87.877 (2)°
*V* = 1186.13 (7) Å^3^
*Z* = 2
Mo *K*α radiation
μ = 0.18 mm^−1^
*T* = 295 K
0.24 × 0.20 × 0.16 mm

#### Data collection

Bruker Kappa APEXII diffractometer
Absorption correction: multi-scan (*SADABS*; Sheldrick, 1996 ▶) *T*_min_ = 0.958, *T*_max_ = 0.972
26965 measured reflections
5046 independent reflections
3632 reflections with *I* > 2σ(*I*)
*R*_int_ = 0.025

#### Refinement

*R*[*F* ^2^ > 2σ(*F* ^2^)] = 0.043
*wR*(*F* ^2^) = 0.124
*S* = 1.03
5046 reflections
310 parameters
H-atom parameters constrained
Δρ_max_ = 0.18 e Å^−3^
Δρ_min_ = −0.29 e Å^−3^

### 

Data collection: *APEX2* (Bruker, 2004 ▶); cell refinement: *SAINT* (Bruker, 2004 ▶); data reduction: *SAINT*; program(s) used to solve structure: *SHELXS97* (Sheldrick, 2008 ▶); program(s) used to refine structure: *SHELXL97* (Sheldrick, 2008 ▶); molecular graphics: *PLATON* (Spek, 2009 ▶); software used to prepare material for publication: *SHELXL97*.

## Supplementary Material

Crystal structure: contains datablocks global, I. DOI: 10.1107/S1600536809026506/bt2993sup1.cif

Structure factors: contains datablocks I. DOI: 10.1107/S1600536809026506/bt2993Isup2.hkl

Additional supplementary materials:  crystallographic information; 3D view; checkCIF report

## Figures and Tables

**Table 1:** Hydrogen-bond geometry (Å, °)

*D*—H⋯*A*	*D*—H	H⋯*A*	*D*⋯*A*	*D*—H⋯*A*
C6—H6⋯O1	0.93	2.56	2.911 (3)	103
C8—H8⋯O2	0.93	2.31	2.894 (3)	121
C11—H11⋯O4	0.93	2.36	2.885 (2)	115
C18—H18*A*⋯O1	0.97	2.23	2.855 (3)	122
C18—H18*B*⋯O3	0.97	2.33	2.930 (3)	119
C25—H25*B*⋯O1^i^	0.96	2.38	3.231 (3)	147
C9—H9⋯O2^ii^	0.93	2.58	3.503 (3)	174

Symmetry codes: (i) 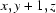; (ii) 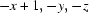.

**Fig. 1.**
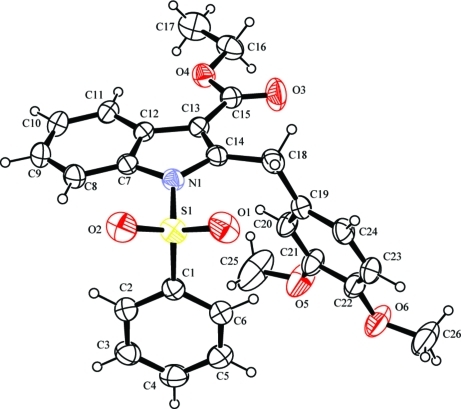
The molecular structure of the title compound, with atom labels and 30% probability displacement ellipsoids for non-H atoms.

**Fig. 2.**
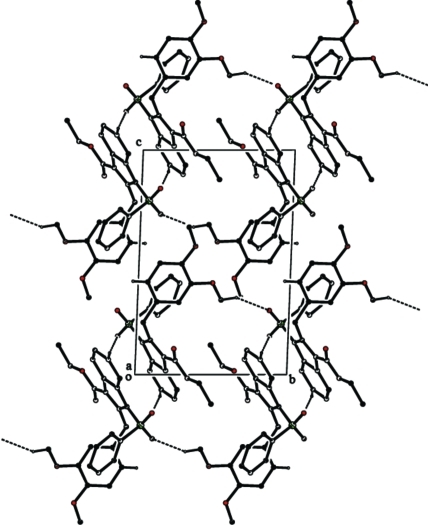
The packing of the title compound, viewed down the a axis. Hydrogen bonds are shown as dashed lines. H atoms not involved in hydrogen bonding have been omitted.

## supplementary crystallographic information

### Comment

The chemistry of indole has been of increasing interest, since several compounds of this type possess diverse biological activities (Macor *et al.*, 1992). In addition, phenylsulfonyl indole compounds inhibit the HIV-1 RT enzyme *in vitro* and HTLVIIIb viral spread in MT-4 human T-lymphoid cells (Williams *et al.*, 1993).

The geometric parameters of the title compound, (I), (Fig. 1) agree well with the reported similar structures (Chakkaravarthi *et al.*, 2007; Chakkaravarthi *et al.*, 2008). The phenyl ring makes a dihedral angle of 82.5 (1)° with the indole ring system. The two aromatic rings C1—C6 and C19—C24 are inclined at an angle of 44.2 (1)° with respect to each other. The sum of the bond angles around N1 [358.8 (5)°] indicate the *sp*^2^ hybridized state. The torsion angles O1—S1—N1—C14 and O2—S1—N1—C7 [27.8 (2) ° and -37.1 (2) °, respectively] indicate the *syn* conformation of the sulfonyl moiety.

A distorted tetrahedral geometry [O1—S1—O2 = 120.4 (1) ° and O1—S1—N1 = 106.9 (1) °] around S1 is observed. The widening of the angles may be due to repulsive interactions between the two short S=O bonds.

The molecular structure is stabilized by weak intramolecular C—H···O interactions and the crystal packing is stabilized by weak intermolecular C—H···O interactions. The C6—H6···O1 interaction generate an S(5) graph set motif and C8—H8···O2 and C11—H11···O4 interactions generate S(6) graph set motifs (Bernstein *et al.*, 1995).

### Experimental

Ethyl 2-(acetoxymethyl)-1-(phenylsulfonyl)-1*H*-indole-3-carboxylate (0.39 g, 0.97 mmol) was dissolved in dry 1,2-dichloroethane (15 ml). To this, anhydrous Ferric chloride (0.02 g, 0.09 mmoL) and 1,2-dimethoxy benzene (0.15 ml, 1.16 mmoL) were added under nitrogen atmosphere. It was refluxed for 5 hr and cooled to room temperature. Ferric chloride was carefully filtered off and the filtrate was poured to water (50 ml) and extracted with chloroform (30 ml). The organic layer was separated and dried over anhydrous sodium sulfate. The solvent was removed under reduced pressure to give the product. It was recrystallized from methanol. Yield: 0.28 g (61%), M.Pt: 134–136°C.

### Refinement

H atoms were positioned geometrically and refined using riding model with C—H = 0.93Å and *U*_iso_(H) = 1.2Ueq(C) for aromatic C—H, C—H = 0.97Å and *U*_iso_(H) = 1.2Ueq(C) for CH_2_, C—H = 0.96Å and *U*_iso_(H) = 1.5Ueq(C) for CH_3_.

### Crystal data

**Table:**

C_26_H_25_NO_6_S	*Z* = 2
*M**_r_* = 479.53	*F*(000) = 504
Triclinic, *P*1	*D*_x_ = 1.343 Mg m^−^^3^
Hall symbol: -P 1	Mo *K*α radiation, λ = 0.71073 Å
*a* = 9.2914 (3) Å	Cell parameters from 5120 reflections
*b* = 9.3008 (3) Å	θ = 2.4–25.1°
*c* = 14.1561 (5) Å	µ = 0.18 mm^−^^1^
α = 87.367 (2)°	*T* = 295 K
β = 76.158 (2)°	Block, colourless
γ = 87.877 (2)°	0.24 × 0.20 × 0.16 mm
*V* = 1186.13 (7) Å^3^	

### Data collection

**Table:**

Bruker Kappa APEX2 diffractometer	5046 independent reflections
Radiation source: fine-focus sealed tube	3632 reflections with *I* > 2σ(*I*)
graphite	*R*_int_ = 0.025
ω and φ scans	θ_max_ = 26.8°, θ_min_ = 1.5°
Absorption correction: multi-scan (*SADABS*; Sheldrick, 1996)	*h* = −11→11
*T*_min_ = 0.958, *T*_max_ = 0.972	*k* = −11→11
26965 measured reflections	*l* = −17→17

### Refinement

**Table:**

Refinement on *F*^2^	Primary atom site location: structure-invariant direct methods
Least-squares matrix: full	Secondary atom site location: difference Fourier map
*R*[*F*^2^ > 2σ(*F*^2^)] = 0.043	Hydrogen site location: inferred from neighbouring sites
*wR*(*F*^2^) = 0.124	H-atom parameters constrained
*S* = 1.03	*w* = 1/[σ^2^(*F*_o_^2^) + (0.0563*P*)^2^ + 0.284*P*] where *P* = (*F*_o_^2^ + 2*F*_c_^2^)/3
5046 reflections	(Δ/σ)_max_ < 0.001
310 parameters	Δρ_max_ = 0.18 e Å^−^^3^
0 restraints	Δρ_min_ = −0.29 e Å^−^^3^

### Fractional atomic coordinates and isotropic or equivalent isotropic displacement parameters (Å^2^)

**Table:**

	*x*	*y*	*z*	*U*_iso_*/*U*_eq_	
S1	0.13534 (6)	−0.05077 (5)	0.22554 (4)	0.06267 (17)	
O4	−0.19660 (14)	0.34677 (15)	−0.01641 (10)	0.0687 (4)	
N1	0.06324 (16)	0.04353 (16)	0.14286 (11)	0.0546 (4)	
O2	0.26358 (17)	−0.12263 (15)	0.17097 (12)	0.0813 (4)	
O1	0.01860 (18)	−0.13039 (16)	0.28540 (12)	0.0863 (5)	
C12	0.05829 (19)	0.2019 (2)	0.01735 (12)	0.0534 (4)	
O5	−0.2124 (3)	0.48992 (18)	0.40802 (13)	0.1186 (7)	
O3	−0.34719 (17)	0.2500 (2)	0.11472 (13)	0.1079 (6)	
C24	−0.3063 (2)	0.0625 (2)	0.41242 (14)	0.0635 (5)	
H24	−0.3268	−0.0350	0.4158	0.076*	
C19	−0.24165 (19)	0.12692 (19)	0.32427 (13)	0.0548 (4)	
C13	−0.08945 (18)	0.1877 (2)	0.07827 (13)	0.0540 (4)	
C1	0.19069 (19)	0.07726 (18)	0.29540 (13)	0.0532 (4)	
C14	−0.08487 (18)	0.09319 (19)	0.15310 (13)	0.0536 (4)	
C2	0.3292 (2)	0.1354 (2)	0.26491 (14)	0.0611 (5)	
H2	0.3928	0.1083	0.2068	0.073*	
C20	−0.2098 (2)	0.2712 (2)	0.32182 (13)	0.0633 (5)	
H20	−0.1652	0.3168	0.2628	0.076*	
C18	−0.2119 (2)	0.0430 (2)	0.23233 (14)	0.0632 (5)	
H18A	−0.1932	−0.0572	0.2489	0.076*	
H18B	−0.3006	0.0477	0.2075	0.076*	
C7	0.15157 (19)	0.11180 (19)	0.05862 (13)	0.0530 (4)	
O6	−0.3423 (2)	0.3687 (2)	0.57147 (12)	0.1124 (7)	
C8	0.3023 (2)	0.0979 (2)	0.01668 (15)	0.0659 (5)	
H8	0.3635	0.0364	0.0443	0.079*	
C15	−0.2254 (2)	0.2614 (2)	0.06311 (15)	0.0637 (5)	
C10	0.2676 (2)	0.2679 (3)	−0.10809 (15)	0.0771 (6)	
H10	0.3086	0.3210	−0.1648	0.092*	
C23	−0.3416 (2)	0.1395 (3)	0.49619 (15)	0.0721 (6)	
H23	−0.3862	0.0937	0.5552	0.087*	
C22	−0.3116 (2)	0.2824 (2)	0.49306 (14)	0.0734 (6)	
C3	0.3721 (2)	0.2339 (2)	0.32159 (16)	0.0724 (6)	
H3	0.4653	0.2739	0.3019	0.087*	
C11	0.1178 (2)	0.2816 (2)	−0.06740 (14)	0.0677 (5)	
H11	0.0577	0.3429	−0.0961	0.081*	
C6	0.0959 (2)	0.1151 (3)	0.38187 (15)	0.0730 (6)	
H6	0.0031	0.0744	0.4024	0.088*	
C21	−0.2426 (3)	0.3481 (2)	0.40464 (15)	0.0719 (6)	
C9	0.3577 (2)	0.1780 (3)	−0.06701 (16)	0.0741 (6)	
H9	0.4584	0.1711	−0.0965	0.089*	
C16	−0.3218 (3)	0.4260 (2)	−0.03882 (19)	0.0812 (6)	
H16A	−0.3649	0.4908	0.0128	0.097*	
H16B	−0.3971	0.3602	−0.0453	0.097*	
C5	0.1408 (3)	0.2136 (3)	0.43708 (17)	0.0882 (7)	
H5	0.0777	0.2405	0.4955	0.106*	
C4	0.2778 (3)	0.2730 (3)	0.40702 (17)	0.0809 (6)	
H4	0.3068	0.3404	0.4449	0.097*	
C17	−0.2667 (3)	0.5085 (3)	−0.1315 (2)	0.1058 (9)	
H17A	−0.1923	0.5732	−0.1241	0.159*	
H17B	−0.3475	0.5627	−0.1484	0.159*	
H17C	−0.2247	0.4433	−0.1820	0.159*	
C25	−0.1090 (5)	0.5491 (3)	0.3324 (2)	0.170 (2)	
H25A	−0.0150	0.5004	0.3284	0.255*	
H25B	−0.1003	0.6493	0.3430	0.255*	
H25C	−0.1390	0.5392	0.2728	0.255*	
C26	−0.4361 (4)	0.3220 (4)	0.65636 (19)	0.1280 (12)	
H26A	−0.5290	0.2974	0.6437	0.192*	
H26B	−0.4524	0.3971	0.7024	0.192*	
H26C	−0.3927	0.2388	0.6826	0.192*	

### Atomic displacement parameters (Å^2^)

**Table:**

	*U*^11^	*U*^22^	*U*^33^	*U*^12^	*U*^13^	*U*^23^
S1	0.0670 (3)	0.0450 (3)	0.0787 (3)	−0.0027 (2)	−0.0224 (2)	−0.0013 (2)
O4	0.0577 (8)	0.0723 (9)	0.0770 (9)	0.0095 (6)	−0.0196 (7)	−0.0045 (7)
N1	0.0505 (8)	0.0554 (8)	0.0594 (9)	−0.0013 (6)	−0.0142 (7)	−0.0106 (7)
O2	0.0858 (10)	0.0559 (8)	0.1053 (12)	0.0212 (7)	−0.0288 (9)	−0.0201 (8)
O1	0.0913 (11)	0.0628 (9)	0.1081 (12)	−0.0266 (8)	−0.0310 (9)	0.0210 (8)
C12	0.0492 (9)	0.0633 (11)	0.0486 (10)	0.0017 (8)	−0.0110 (7)	−0.0180 (8)
O5	0.183 (2)	0.0696 (10)	0.0809 (11)	−0.0411 (12)	0.0196 (12)	−0.0157 (9)
O3	0.0499 (8)	0.1632 (18)	0.0973 (12)	0.0196 (10)	0.0004 (8)	0.0213 (12)
C24	0.0580 (11)	0.0606 (11)	0.0689 (13)	−0.0168 (9)	−0.0097 (9)	0.0126 (10)
C19	0.0467 (9)	0.0563 (10)	0.0592 (11)	−0.0086 (8)	−0.0079 (8)	0.0014 (8)
C13	0.0466 (9)	0.0643 (11)	0.0515 (10)	0.0006 (8)	−0.0106 (7)	−0.0143 (9)
C1	0.0537 (10)	0.0507 (10)	0.0561 (10)	0.0013 (8)	−0.0166 (8)	0.0045 (8)
C14	0.0486 (9)	0.0563 (10)	0.0577 (10)	−0.0051 (8)	−0.0130 (8)	−0.0162 (8)
C2	0.0586 (11)	0.0645 (11)	0.0599 (11)	−0.0039 (9)	−0.0135 (9)	−0.0014 (9)
C20	0.0734 (12)	0.0599 (11)	0.0489 (10)	−0.0141 (9)	0.0015 (9)	0.0043 (8)
C18	0.0546 (10)	0.0623 (11)	0.0725 (12)	−0.0128 (9)	−0.0123 (9)	−0.0068 (9)
C7	0.0496 (9)	0.0565 (10)	0.0540 (10)	−0.0004 (8)	−0.0112 (8)	−0.0185 (8)
O6	0.1424 (17)	0.1171 (14)	0.0607 (10)	−0.0368 (12)	0.0179 (10)	−0.0204 (9)
C8	0.0511 (10)	0.0770 (13)	0.0694 (13)	0.0075 (9)	−0.0123 (9)	−0.0190 (10)
C15	0.0533 (11)	0.0792 (13)	0.0597 (12)	0.0057 (9)	−0.0148 (9)	−0.0144 (10)
C10	0.0677 (13)	0.1025 (17)	0.0533 (11)	−0.0056 (12)	0.0022 (10)	−0.0074 (11)
C23	0.0682 (12)	0.0859 (15)	0.0555 (12)	−0.0210 (11)	−0.0023 (9)	0.0174 (11)
C22	0.0777 (14)	0.0842 (15)	0.0511 (11)	−0.0149 (11)	0.0008 (10)	−0.0037 (10)
C3	0.0688 (13)	0.0759 (13)	0.0768 (14)	−0.0156 (10)	−0.0245 (11)	0.0005 (11)
C11	0.0611 (11)	0.0893 (15)	0.0502 (11)	0.0036 (10)	−0.0086 (9)	−0.0067 (10)
C6	0.0588 (11)	0.0993 (16)	0.0610 (12)	−0.0080 (11)	−0.0135 (9)	−0.0048 (11)
C21	0.0886 (15)	0.0598 (12)	0.0582 (12)	−0.0186 (10)	0.0031 (10)	−0.0015 (9)
C9	0.0508 (10)	0.0985 (16)	0.0684 (13)	0.0021 (11)	−0.0018 (10)	−0.0234 (12)
C16	0.0718 (13)	0.0725 (14)	0.1077 (18)	0.0176 (11)	−0.0386 (13)	−0.0152 (13)
C5	0.0776 (15)	0.124 (2)	0.0647 (13)	0.0054 (14)	−0.0168 (11)	−0.0265 (13)
C4	0.0899 (16)	0.0852 (15)	0.0768 (15)	−0.0024 (13)	−0.0356 (13)	−0.0175 (12)
C17	0.122 (2)	0.0814 (17)	0.124 (2)	0.0123 (16)	−0.0544 (19)	0.0124 (16)
C25	0.293 (5)	0.085 (2)	0.100 (2)	−0.092 (3)	0.029 (3)	0.0008 (17)
C26	0.153 (3)	0.151 (3)	0.0598 (15)	−0.021 (2)	0.0180 (17)	−0.0110 (16)

### Geometric parameters (Å, °)

**Table:**

S1—O1	1.4160 (15)	O6—C26	1.368 (3)
S1—O2	1.4182 (15)	O6—C22	1.368 (3)
S1—N1	1.6809 (16)	C8—C9	1.371 (3)
S1—C1	1.7485 (18)	C8—H8	0.9300
O4—C15	1.326 (2)	C10—C9	1.369 (3)
O4—C16	1.445 (2)	C10—C11	1.377 (3)
N1—C14	1.411 (2)	C10—H10	0.9300
N1—C7	1.414 (2)	C23—C22	1.365 (3)
C12—C11	1.387 (3)	C23—H23	0.9300
C12—C7	1.392 (3)	C22—C21	1.388 (3)
C12—C13	1.443 (2)	C3—C4	1.369 (3)
O5—C21	1.363 (3)	C3—H3	0.9300
O5—C25	1.365 (3)	C11—H11	0.9300
O3—C15	1.196 (2)	C6—C5	1.369 (3)
C24—C19	1.370 (2)	C6—H6	0.9300
C24—C23	1.379 (3)	C9—H9	0.9300
C24—H24	0.9300	C16—C17	1.479 (4)
C19—C20	1.382 (2)	C16—H16A	0.9700
C19—C18	1.511 (3)	C16—H16B	0.9700
C13—C14	1.353 (3)	C5—C4	1.370 (3)
C13—C15	1.471 (3)	C5—H5	0.9300
C1—C6	1.378 (3)	C4—H4	0.9300
C1—C2	1.378 (3)	C17—H17A	0.9600
C14—C18	1.492 (3)	C17—H17B	0.9600
C2—C3	1.375 (3)	C17—H17C	0.9600
C2—H2	0.9300	C25—H25A	0.9600
C20—C21	1.367 (3)	C25—H25B	0.9600
C20—H20	0.9300	C25—H25C	0.9600
C18—H18A	0.9700	C26—H26A	0.9600
C18—H18B	0.9700	C26—H26B	0.9600
C7—C8	1.388 (2)	C26—H26C	0.9600
			
O1—S1—O2	120.38 (10)	C11—C10—H10	119.2
O1—S1—N1	106.84 (9)	C22—C23—C24	120.41 (18)
O2—S1—N1	105.33 (9)	C22—C23—H23	119.8
O1—S1—C1	108.93 (9)	C24—C23—H23	119.8
O2—S1—C1	108.65 (9)	C23—C22—O6	125.20 (19)
N1—S1—C1	105.74 (8)	C23—C22—C21	118.95 (19)
C15—O4—C16	116.22 (16)	O6—C22—C21	115.85 (19)
C14—N1—C7	108.32 (15)	C4—C3—C2	120.0 (2)
C14—N1—S1	127.51 (13)	C4—C3—H3	120.0
C7—N1—S1	122.95 (12)	C2—C3—H3	120.0
C11—C12—C7	119.17 (17)	C10—C11—C12	118.6 (2)
C11—C12—C13	133.86 (18)	C10—C11—H11	120.7
C7—C12—C13	106.97 (16)	C12—C11—H11	120.7
C21—O5—C25	117.9 (2)	C5—C6—C1	118.8 (2)
C19—C24—C23	121.22 (18)	C5—C6—H6	120.6
C19—C24—H24	119.4	C1—C6—H6	120.6
C23—C24—H24	119.4	O5—C21—C20	124.45 (18)
C24—C19—C20	118.07 (17)	O5—C21—C22	115.43 (18)
C24—C19—C18	120.49 (17)	C20—C21—C22	120.12 (19)
C20—C19—C18	121.41 (16)	C10—C9—C8	121.29 (19)
C14—C13—C12	109.01 (16)	C10—C9—H9	119.4
C14—C13—C15	124.31 (16)	C8—C9—H9	119.4
C12—C13—C15	126.68 (17)	O4—C16—C17	107.4 (2)
C6—C1—C2	121.12 (18)	O4—C16—H16A	110.2
C6—C1—S1	119.04 (15)	C17—C16—H16A	110.2
C2—C1—S1	119.81 (14)	O4—C16—H16B	110.2
C13—C14—N1	108.19 (15)	C17—C16—H16B	110.2
C13—C14—C18	127.54 (17)	H16A—C16—H16B	108.5
N1—C14—C18	124.23 (17)	C6—C5—C4	120.5 (2)
C3—C2—C1	119.09 (19)	C6—C5—H5	119.7
C3—C2—H2	120.5	C4—C5—H5	119.7
C1—C2—H2	120.5	C3—C4—C5	120.4 (2)
C21—C20—C19	121.20 (17)	C3—C4—H4	119.8
C21—C20—H20	119.4	C5—C4—H4	119.8
C19—C20—H20	119.4	C16—C17—H17A	109.5
C14—C18—C19	115.27 (15)	C16—C17—H17B	109.5
C14—C18—H18A	108.5	H17A—C17—H17B	109.5
C19—C18—H18A	108.5	C16—C17—H17C	109.5
C14—C18—H18B	108.5	H17A—C17—H17C	109.5
C19—C18—H18B	108.5	H17B—C17—H17C	109.5
H18A—C18—H18B	107.5	O5—C25—H25A	109.5
C8—C7—C12	121.86 (18)	O5—C25—H25B	109.5
C8—C7—N1	130.62 (18)	H25A—C25—H25B	109.5
C12—C7—N1	107.51 (15)	O5—C25—H25C	109.5
C26—O6—C22	119.5 (2)	H25A—C25—H25C	109.5
C9—C8—C7	117.6 (2)	H25B—C25—H25C	109.5
C9—C8—H8	121.2	O6—C26—H26A	109.5
C7—C8—H8	121.2	O6—C26—H26B	109.5
O3—C15—O4	122.87 (19)	H26A—C26—H26B	109.5
O3—C15—C13	126.1 (2)	O6—C26—H26C	109.5
O4—C15—C13	111.03 (16)	H26A—C26—H26C	109.5
C9—C10—C11	121.5 (2)	H26B—C26—H26C	109.5
C9—C10—H10	119.2		
			
O1—S1—N1—C14	27.85 (17)	C14—N1—C7—C8	−178.86 (17)
O2—S1—N1—C14	156.95 (14)	S1—N1—C7—C8	12.9 (3)
C1—S1—N1—C14	−88.10 (15)	C14—N1—C7—C12	−0.29 (18)
O1—S1—N1—C7	−166.29 (13)	S1—N1—C7—C12	−168.52 (11)
O2—S1—N1—C7	−37.18 (15)	C12—C7—C8—C9	0.8 (3)
C1—S1—N1—C7	77.77 (14)	N1—C7—C8—C9	179.23 (17)
C23—C24—C19—C20	1.3 (3)	C16—O4—C15—O3	−0.6 (3)
C23—C24—C19—C18	−176.42 (18)	C16—O4—C15—C13	−179.67 (16)
C11—C12—C13—C14	179.75 (19)	C14—C13—C15—O3	1.4 (3)
C7—C12—C13—C14	0.14 (19)	C12—C13—C15—O3	−179.1 (2)
C11—C12—C13—C15	0.2 (3)	C14—C13—C15—O4	−179.51 (16)
C7—C12—C13—C15	−179.39 (16)	C12—C13—C15—O4	−0.1 (3)
O1—S1—C1—C6	−19.05 (18)	C19—C24—C23—C22	−0.5 (3)
O2—S1—C1—C6	−151.89 (16)	C24—C23—C22—O6	179.8 (2)
N1—S1—C1—C6	95.47 (16)	C24—C23—C22—C21	−1.2 (3)
O1—S1—C1—C2	159.04 (15)	C26—O6—C22—C23	−14.4 (4)
O2—S1—C1—C2	26.20 (17)	C26—O6—C22—C21	166.6 (3)
N1—S1—C1—C2	−86.45 (16)	C1—C2—C3—C4	0.0 (3)
C12—C13—C14—N1	−0.32 (19)	C9—C10—C11—C12	0.2 (3)
C15—C13—C14—N1	179.22 (16)	C7—C12—C11—C10	0.3 (3)
C12—C13—C14—C18	−178.34 (16)	C13—C12—C11—C10	−179.24 (19)
C15—C13—C14—C18	1.2 (3)	C2—C1—C6—C5	0.9 (3)
C7—N1—C14—C13	0.38 (18)	S1—C1—C6—C5	178.94 (18)
S1—N1—C14—C13	167.92 (12)	C25—O5—C21—C20	−18.9 (5)
C7—N1—C14—C18	178.48 (15)	C25—O5—C21—C22	161.3 (3)
S1—N1—C14—C18	−14.0 (2)	C19—C20—C21—O5	179.0 (2)
C6—C1—C2—C3	−0.7 (3)	C19—C20—C21—C22	−1.2 (3)
S1—C1—C2—C3	−178.79 (15)	C23—C22—C21—O5	−178.2 (2)
C24—C19—C20—C21	−0.5 (3)	O6—C22—C21—O5	0.9 (3)
C18—C19—C20—C21	177.3 (2)	C23—C22—C21—C20	2.0 (4)
C13—C14—C18—C19	−93.4 (2)	O6—C22—C21—C20	−178.9 (2)
N1—C14—C18—C19	88.8 (2)	C11—C10—C9—C8	−0.2 (3)
C24—C19—C18—C14	−152.33 (18)	C7—C8—C9—C10	−0.3 (3)
C20—C19—C18—C14	30.0 (3)	C15—O4—C16—C17	−177.62 (19)
C11—C12—C7—C8	−0.9 (3)	C1—C6—C5—C4	−0.3 (4)
C13—C12—C7—C8	178.82 (16)	C2—C3—C4—C5	0.6 (4)
C11—C12—C7—N1	−179.58 (15)	C6—C5—C4—C3	−0.4 (4)
C13—C12—C7—N1	0.10 (18)		

### Hydrogen-bond geometry (Å, °)

**Table:**

*D*—H···*A*	*D*—H	H···*A*	*D*···*A*	*D*—H···*A*
C6—H6···O1	0.93	2.56	2.911 (3)	103
C8—H8···O2	0.93	2.31	2.894 (3)	121
C11—H11···O4	0.93	2.36	2.885 (2)	115
C18—H18A···O1	0.97	2.23	2.855 (3)	122
C18—H18B···O3	0.97	2.33	2.930 (3)	119
C25—H25B···O1^i^	0.96	2.38	3.231 (3)	147
C9—H9···O2^ii^	0.93	2.58	3.503 (3)	174

Symmetry codes: (i) *x*, *y*+1, *z*; (ii) −*x*+1, −*y*, −*z*.
